# Absorbing Power of Wounds

**Published:** 1880-05

**Authors:** 


ABSORBING POWER OF WOUNDS.

M. Maas, of Fribourg, has made a series of researches on the absorbing power of wounds, and the results which he has arrived at are partly opposed to the opinion admitted up to the present. A wound cauterized with the hot iron, nitrate of silver or nitric acid, absorbs like an intact wound; the absorption is much more rapid if the wound has been in contact with carbolic acid, as in Lister's dressing; it is nil in cases of cauterization with chlorate of zinc. In wounds treated openly, a crust is formed which at the end of six hours is unpermeable; it becomes so only at the end of three days if the wound has previously been covered over with a wet dressing.—*Le Prog. Med.*

